# Sergei Prokofiev’s Children’s Pieces, Op. 65: a comprehensive approach to learning about a composer and his works: biography, style, form and analysis

**DOI:** 10.1186/2193-1801-3-23

**Published:** 2014-01-13

**Authors:** Peter Daniel Klein

**Affiliations:** Gyeongnam National University of Science and Technology, Gyeongnam-do, Jinju-si, Dongjin-ro, 33 660-758 South Korea

## Abstract

This article is written for the benefit of piano teachers and students, but can be of benefit to any music teacher or student. It is a case study using Prokofiev’s lesser known pedagogical work for the piano, which serves as an example of information gathering to apply toward a more effective method of instruction, which requires the teacher and student to exhaustively examine both composer and music in order to exact a more artistic, accurate performance.

Much of the interpretation is based on Prokofiev’s own thoughts as expressed in his personal memoirs and from his most distinguished music critics, many of whom were his peers during his lifetime, while some is taken from common sources, which are readily available to teacher and student. It is my belief that it is possible to divine extraordinary interpretations, information and outcomes from common sources.

As the student and teacher gather information, it can be used to determine what should be included in a performance based not only on the composer’s explicit directions, but also on implicit information that could lead to an inspired, original interpretation. It is written with the belief that music is more than the dots and lines on the page and that teaching and learning must be approached with that in mind. It is hoped that once teacher and student have completed this case study, the method will transfer to all future musical endeavors.

## A biography tracing stylistic and technical ability

When composers reach a status of fame and acceptance, one might argue that it is the tendency of historians and biographers to imbue genius on all that they do, no matter how mundane. The compositions are presented as faultless and the facility at their instrument becomes extraordinary, almost Herculean and even the errors become “charming.” The road to success becomes a matter of inevitability, almost on the order of predestination. However, we should take into account the struggle of the musician for skill and acceptance, since it most likely shapes each and every work. Time and circumstance may be as much a teacher as any music instructor and its impact is sometimes more profound.

Therefore, I intend to use Prokofiev’s biography, along with style, form, and analysis in an instructive fashion to aid in analyzing his lesser known ***Children’s Pieces****,* Op. 65 for piano. I believe this can assist piano teachers in providing comprehensive instruction which uses the lessons and experiences of the composer, in part, as lessons to guide us to an informed performance. Some easy access references, such as the ***New Grove Dictionary of Music*** will be utilized for the benefit of younger students. Common references can inform performance as well as rare, scholarly material and students should be taught to access the reference section of the library every time they are assigned a new piece. The assignment can be broken into four parts: biography, style, form and analysis, although the final two facets might be combined since they are so closely related. If a teacher models this behavior and requires it of students, later his or her students will follow suit and dig deeper, eventually becoming performing scholars.

Lets us begin by examining Prokofiev’s biographical information with the end goal of applying it to the piece of music in question. Among other things, this section can particularly inform the teacher and student as to why Prokofiev often requires performers to do things that could be characterized as percussive, shocking and un-pianistic. This section helps the teacher and student understand that it would be wrong to mitigate these elements in a performance. This section can also be inspiring to students of great talent who struggle with technique and marginal grades to push forward and eventually succeed. It can also remind teachers not to underestimate students and to be patient and persistent with them. So let us proceed with the biographical section.

Without a doubt, in the case of Prokofiev, the element of innate talent was certain early in his childhood. His predilection toward composition was apparent and he studied beginning harmony and composition under the composer Rheinhold Glier (Prokofiev, 1979a, p 30). His talent as a pianist was evident while his achievements until later were questionable. He was hardly a child prodigy. It took years for him to even approach performance competence at the keyboard (The New Grove Dictionary of Music and Musicians, 2001a, p. 289). His award of the Rubinstein Prize at the conservatory was more a publicity stunt than a legitimate victory (The New Grove Dictionary of Music and Musicians, 2001b, p. 290). From this triumph Prokofiev learned early to exploit the sensational in order to gain sufficient attention and attract success. With competitors on the order of Igor Stravinsky and Sergei Rachmaninov and the emerging world of atonality and its sheer shock value, self-promotion was indispensable to his success (The New Grove Dictionary of Music and Musicians, 2001c, pp. 291-292).

Prokofiev began his piano lessons as a child. They were administered by his mother who was a well-educated woman and a good pianist. She practiced up to six hours a day (Prokofiev, 1979b, p. 13). His father was a wealthy agricultural engineer and landowner. The family lived on an isolated rural estate in Sontsovska in the Ukraine. The young Prokofiev was a pampered and spoiled only child usually wanting for nothing. Attention was lavished on him and during his mother’s teaching he was exposed to Beethoven, Chopin, and Anton Rubinstein (Prokofiev, 1979c, pp. 4-13). Soon, the effort to instill musical ability and appreciation in him gave birth to his flair for composition. It began to flower especially under the tutelage of Glier. At age 12, some of his compositions, including a piano and violin sonata, were performed for composer Sergei Taneyev on a trip to Moscow, and on another trip in 1904, composer Alexander Glazunov recommended application to the conservatory in St. Petersburg. The family was hesitant to send him so far from home at such a young age, but upon passing the entrance exams, he was admitted to the conservatory and the matter was settled (The New Grove Dictionary of Music and Musicians, 2001d, p. 288).

The times during his schooling were far from serene. In 1905 the stirrings of the eventual Russian Revolution were being felt and, with the discord, the conservatory was constantly in flux. The faculty was always shifting and the conservatory itself was often closed, leaving the students to be taught in the instructor’s home gratis. Anatoly Lyadov was the composition and harmony instructor and he was very lazy during this period, especially without payment. Lessons were not individual and the atmosphere was unchallenging (Prokofiev, 1979d, pp. 94-101). Lyadov was difficult and disliked Prokofiev’s modern leanings. In fact, in one lesson to a dozen students he stated: “If I had money, do you really think I would be teaching you? Except for maybe two or three of you, you won’t become composers in any case (Prokofiev, 1979e, p. 105).” He thought Prokofiev lazy and sloppy, but later it turns out that in spite of this he still believed Prokofiev and Nikolai Miaskovsky to be two of the three.

Prokofiev disliked Lyadov and considered his teaching boring and uninspiring. So, he corresponded to his father about this problem. His father responded with a letter requesting individual lessons which were accompanied by payment (Prokofiev, 1979f, p. 93). The atmosphere soon changed. The young composer was treated with greater care. Prokofiev still complained about Lyadov’s difficult nature and request for simplicity in all compositions. Each time he presented a piece he thought was ingenious, he was told to alter it and simplify it (Prokofiev, 1979g, p. 96). Perhaps this is where his tendency toward a thin harmonic, polyphonic style began taking shape. His style of orchestration can be attributed in part to Nikolai Rimsky-Korsakov, although he rebelled against some of that instruction too (Krebs, 1970a, p. 140). The other great influence on his style of orchestration was that of the modernist Alexander Tcherepnin by whom he was taught conducting. Tcherepnin encouraged Prokofiev to experiment with instrumental sounds. As a result, color became a unique tool for him (The New Grove Dictionary of Music and Musicians, 2001a, p. 289).

While his experience thus far at the conservatory was comprised mainly of theory and composition, his pianistic abilities suffered. He had only taken obligatory piano and he hoped to enter into individual study. He owed a great debt to his professor Alexander Winkler. Winkler was a good theoretician and musician, but was not exceptional. He suffered from such incredible stage fright, that it sometimes marred his performances. Yet, when all others failed to push Prokofiev into private study, it was Winkler who was instrumental in sponsoring him. However, the lessons he received from Winkler, and the childhood lessons from his mother, still left his technical abilities extremely lacking. He had developed his own style and dexterity, but hated the discipline of practice and could not play any classical repertory. In any case, he undoubtedly still benefited from Winkler’s instruction (Prokofiev, 1979h, pp. 88-89, 92).

One night he had a stroke of luck. At a recital, during which he played Chopin’s ***Etude No. 1***, Brahms’s ***Rhapsody in G minor***, and Rubinstein’s ***C Major Etude No. 1***, the famed piano pedagogue and performer Anna Esipova attended and was smiling as he performed. One of her students, Boris Stepanovich Zakharov, approached Prokofiev advising him to attempt studies with her, which was nearly impossible. He was reassured by Zakharov that she would admit him. In his memoir, Prokofiev recalled Winkler was beaming with joy after the recital. As much as he hoped to study with Esipova, he had a deep loyalty toward Winkler and felt as if a switch would be a betrayal (Prokofiev, 1979i, p. 158).

The day after the recital, his close friend Nikolai Miaskovsky urged him to change instructors. When Prokofiev protested about betraying Winkler, Miaskovsky retorted, “When you’re marching toward your goal, you mustn’t look at the corpses you must walk over (Prokofiev, 1979j, p. 159).” As Prokofiev began evaluating the idea of change, he became enamored with it. As he became more inclined to act on the impulse, he wrote to his father to propose it. His father, who felt the same gratitude toward Winkler, protested, which caused Prokofiev to quote Miaskovsky’s retort. His father wrote back, “But the trouble is, the corpses sometimes rise up and club you on the back of the head (Prokofiev, 1979j, p. 159).”

Eventually, as time progressed, Prokofiev began performing his own works which received quite a bit of publicity. His works were labeled ‘unintelligible’ and ‘ultra-modern.’ He saw the benefit of this exposure and did his best to cater to that same sensational image mentioned earlier, of the enfant terrible (The New Grove Dictionary of Music and Musicians, 2001a, p. 289). With this new found attention, and the charming cajoling of her student Zakharov, who was Prokofiev’s friend, Esipova agreed to take Prokofiev as a student, but not without gaining the permission of Winkler prior to admission. She knew Prokofiev was his favorite student. Prokofiev finally approached Winkler, feeling like a traitor, and was given permission to switch at the end of his current term in 1909 (Prokofiev, 1979k, p. 169).

In 1909, at age 18, Prokofiev graduated from the conservatory with a degree in composition, with no better than average grades. After graduation, he continued to study piano and conducting, reasoning that performances of his own music would be the sole means to achieving his success. So, in 1909, after graduation, as he embarked on a second degree, he began piano courses with Esipova and continued until 1914 (Nest’ev, 1960a, p. 39). It was not long before he began rebelling against her demanding requirements. He hated the classical repertoire and the compulsory exercises. Esipova would not tolerate his rebellion and threatened him with expulsion. Only because of that threat did he begin to comply. His four years of study with Esipova were the turning point in the evolution of his pianistic abilities. He achieved greater technical prowess and even learned to play, and value, Mozart and Schubert (Nest’ev, 1960b, p. 37). It is quite probable that this focus on classical composers had a great impact on his neo-classical compositional style, in addition to the early guidance of Glier and the conservatory instruction provided by Lyadov.

In 1914, he again graduated from the conservatory with a degree in piano. As mentioned in the introduction, he did so while winning the Rubinstein prize with the impudence of performing his own composition, instead of, as tradition held, by performing an accepted piece of standard repertory. Though he had made progress with Esipova, it is doubtful that he would have been able to compete on any other terms and it is unlikely that he could have survived the competition against the superior pianists participating. Ultimately, he was able to achieve this first success, but only by transforming a piano competition into a composition contest. This was in line with the strategy for which he had prepared educationally and it was a boon.

His tactics turned the piano competition upside-down. At first, the judges protested the breech of tradition. Many voted to disqualify him insisting on a standard piece from the repertory. The resulting vote from the judges was closely divided and was not even nearly unanimous (Nest’ev, 1960c, p. 290. “‘…the older professors, headed by Glazounov, the director of the Conservatoire, voted against.’” “The anecdote reveals an artist who is eager to participate in a traditional activity, but insists on doing so on his own terms. It is reminiscent of the play of tradition and innovation (Minturn, 1997a, p. 74).” Again we see in greater detail how with Prokofiev the sensational prevails, yet the ***First Piano Concerto*** is a fine work and is still standard repertory. But this is in keeping with what Miaskovsky referred to as Prokofiev’s “fiery temperament and…purely external technical virtues (Prokofiev, 1979l, p. 175).” This is clearly indicative of the early style of Prokofiev and should be reflected in performance of his music, especially that of the early period.

The next few years of the Russian Revolution’s turmoil forced Prokofiev out of the country intermittently until he ended up permanently in France. He had established himself as a composer and pianist in Russia and traveled to London, the United States, and Europe. His exposure to Igor Stravinsky inspired the ***Scythian Suite*** and he spent time with Les Six, especially French composer Francis Poulenc, but while he absorbed some features of their style, he was not in complete agreement with any of them (Krebs, 1970b, pp. 140-42). During this time he found success composing ballets and his relationship with Dyagilev and the Ballet Russe created artistic avenues and built revenues. One particular opera called ***The Steel Step***, which was supposed to promote the Bolshevik ideal of industrialization, was a great success in Paris. In Russia, it was rejected because it was composed using Les Six constructivist ideas and emulates Stravinsky’s ***Rite of Spring*** stylistically (The New Grove Dictionary of Music and Musicians, 2001e, p. 292-293). Also, in this period his love of writing opera grew, however, his success in that venue dwindled. But, despite his failures, he continued to compose in that vein. His opera ***The Fiery Angel*** became an obsession, although production was elusive and his popularity in Europe and elsewhere abroad began to fade.

As this was happening, in 1927, Soviet Russia began attempting to recapture scattered artists who fled after the revolution. Prokofiev was courted by high ranking Soviet officials and it is reported that he negotiated his terms of return with none other than Stalin himself (Krebs, 1970c, p. 151). While Prokofiev was not a man with firm political convictions, he did love adulation and when that was fading in the west, it was plentiful in Russia (Krebs, 1970d, p. 152). Although he was torn between his homeland and Paris, he finally agreed to a return. Eventually, by 1936, when he felt reassured, he moved his family to Moscow. He had been sheepish about his homecoming and for some time kept two homes, one in Moscow and one in Paris.

In Moscow, it seems he had been granted artistic freedom and for a time was immune to political censure. This immunity was short lived and it is evident that his style was to change drastically upon his return. He was expected to produce art accessible by the common man, which was representative of Soviet realism. Although he had not felt any censure upon his return, all modern composers were being denounced and removed from programs (The New Grove Dictionary of Music and Musicians, 2001f, p. 295). Dimitri Shostakovich was publicly denounced and this was when Prokofiev retreated and began working on many small pieces, some for children. This is when the opus 65, ***Music for Children,*** and opus 68, ***Three Children’s Songs*** were written. Other larger works exhibiting the stylistic shift to a more lyrical genre were ***Romeo and Juliet*** and ***Peter and the Wolf*** (The New Grove Dictionary of Music and Musicians, 2001f, p. 295). Stanley Krebs suggests that work with children was often assigned to political offenders and this is why so often Prokofiev worked with youth until his death (Krebs, 1970e, p. 163).

The rest of his life he was both persecuted and praised. Many of his works were banned from performance as Bourgeois and unworthy. His Spanish born wife was arrested in 1939 for spying and sentenced to 20 years in a concentration camp and he was assigned a new young wife, Mira Mendelson, who had communist party affiliations. In 1941, he suffered a heart attack and his health was weakened by recurrences until his death at 62 in 1953. This health problem slowed his compositional progress. So did the trappings of Soviet politics and art censorship. Ironically, at one point, he was denounced by Dimitri Shostakovich who had suffered the same denunciation. He was also attacked by Dimitri Kabalevsky, a composer for whom Prokofiev had no musical respect whatsoever. At one point in time, Kabalevsky was permanently assigned to monitor his work for Soviet musical realism. Kabalevsky believed he was caring for an ill genius (Krebs, 1970f, p. 159-162). Paradoxically on March 5, 1953 both Prokofiev and Stalin died on the same day of the same cause, a cerebral hemorrhage (Robinson, 2002a, pp. 1-3).

The things that one must remember above all regarding Prokofiev’s early experiences were his impetuosity, arrogance, sarcasm, innocence, rebellion, impudence, and disregard for tradition (Krebs, 1970g, p. 142). We should also remember his unbridled ambition and his need to prove his uniqueness that served to shape his compositional style. In fact, I assert that these qualities are present in every note of each composition and could serve as an excellent general description of his musical style. His lack of pianistic training and his brash personality led him to write music in a steely, percussive, un-pianistic style. In that respect, I believe his ignorance and rebellion brought about a novel innovation. In the end, our conclusions in connection to his biography should inform us to remember that the aforementioned qualities should color every performance of his work to varying degrees based on whether early or late.

Finally, Prokofiev has been described as the twentieth century Alexander Borodin and might possibly have reached the level of a Modest Mussorgsky had he not been restricted by politics and beset by personal problems. Renowned Soviet musicologist Stanley Krebs sums up Prokofiev’s accomplishments by saying, “…although his position on the concert stage is assured, Prokofiev left little to excite today’s aspirant composer. No crueler thing could be said, for this was the point of much of his life’s struggles, a success that transcends the concert hall or opera theater (Krebs, 1970h, p. 164).”

## Stylistic periods

It is difficult to properly research and analyze style in such a recent and controversial composer as Prokofiev. When reading the available resources, valid analysis free of political rhetoric is hard to find. Yet, while it is distracting and accounts are more than likely distorted by the scholars, like Bakst and Nest’ev to suit their particular cause, politics were a driving force in shaping his later music. It is suggested by most sources that Prokofiev was apolitical, but I tend to believe this is only partially true. He did love his homeland and although he found artistic haven in other countries, he never felt at home in them. He could never reconcile completely with the styles of the times or countries other than Russia and this is attested to by his own statements. He took elements of many styles and combined them into his own unique, Russian musical voice (Nest’ev, 1960d, p. 459).

I assert that in the late twenties and early thirties, the idealism of communism looked plausible and appealed to a rebellious young man and iconoclast (Krebs, 1970i, pp. 151-152). It seems to me he was taken with the idea of proving a certain kind of Russian artistic superiority and producing an art for the Russian masses (Krebs, 1970j, p. 154). Such a thing, if done successfully, would assure him of leaving the artistic legacy he longed to impress on music history. As Stanley Krebs points out, Prokofiev “…appeared in print as early as 1934 with arch-doctrinaire statements about the need for and production of music for the broad masses, and even characterized the sort of music as ‘light serious’ or ‘serious light’ (Krebs, 1970j, p. 154).”

The fact that Prokofiev had coined terms such as ‘light serious’ to publicly describe a genre of ‘light serious music for the masses’ surely proves that he supported the idea and gave it great consideration. Krebs, who separates the ***Children’s Music*** for piano, ***Peter and the Wolf***, and ***Romeo and Juliet*** from this particular category, does so contrary to his statement about working with children as a communist political punishment (Krebs, 1970k, p. 155). Nest’ev (1960e, p. 488) and Bakst (1966a, p. 304) counter that these types of music were the height of the effort to appeal to the common man in the Soviet ideal. In my opinion, the styles of all of these works are decidedly similar and fall into a ‘light serious’ category that are very appealing to the average person. The production of so many of these types of works in succession surely coincide with the aforementioned government pressure at that particular point in time to produce music for the masses. Prokofiev’s statements about the issue also came at the same time (Nest’ev, 1960f, p.154).

The first years of Prokofiev’s life, he devised a plan to map his road to fame by writing and performing his own, radical, innovative style of composition. He knew it would only work if he were his own greatest proponent, with his percussive, steely style of presentation. In form and harmony, he was a neo-classicist. He believed the sonata form to be the ultimate elastic musical vehicle, but in his early work he was not interested in further developing traditions, he preferred to turn tradition on its ear (Nest’ev, 1960g, p. 461). As mentioned previously, he used many techniques popular in his day as they suited him, but Schoenberg’s atonality was particularly repulsive to him. He felt it cold, mathematical, and excessively rational. He soundly rejected it (Nest’ev, 1960h, p. 455). In Prokofiev’s estimation, though he respected Stravinsky, he felt that he was not a man of deep feeling and that he was more of an observer that was absorbed in his own music (Nest’ev, 1960h, p. 455). His time in France availed him close connections to the French expressionists, but he rejected the thick harmonies for economical ones as he had been trained to do during his studies with Lyadov.

When he was abroad during his first period, he made it very clear that he would adopt many different techniques to accomplish what suited him. It was his belief that if an artist formulates a single logical formula under which to operate, he limits himself (Nest’ev, 1960h, p.455). He felt the need to be an original thinker and to follow his own ideas. He was never satisfied to do things merely because the rules said so (Nest’ev, 1960i, p. 456). He explains it himself in the November 1918 edition of *The Musical Observer*. “When I first left the Conservatory, I had too many ideas and not enough technique to express them as I wished. But I decided this was better than the reverse…I am not ashamed to say that essentially I am a pupil of my own ideas. In all that I write, I have two leading principles—clarity in the expression of my ideas and economy of expression, the avoidance of everything superfluous in expressing them (As cited in Nest’ev, 1960i, p. 456)^a^.”

Prokofiev’s move back to The Soviet Union is the change that marks the second stylistic period in his life. It was a departure from his earlier style to a degree, although he maintained an element of his trademark sound in melody, certain harmonies, and orchestrations (Bakst, 1966b, p. 297). But, I propose that he never departed from the course of becoming a legend, especially if it meant becoming a historical, musical, and ideological icon to the people of his homeland, even if it meant altering his artistic preferences and ideals to accomplish this end. He believed in his homeland, himself, and Russia’s leading role in contemporary music (Nest’ev, 1960j, p. 458).

In my estimation, this was the momentum that drove him to adopt, at times, the lyrical style which he qualified as out and out emotionalism and bad taste in the music of Rachmaninov and Tchaikovsky. The lyricism he detested in Tchaikovsky, Rachmaninov, Medtner, and Scriabin, he overcame (Nest’ev, 1960k, p. 465). He never became a romantic in any true sense of the word, and to Soviets romanticism was decadent and bourgeois, so they will term anything sounding romantic as lyrical, unless it is meant to be derogatory. It then logically becomes a matter of semantics as to what a broad stroke of lush harmony and beautiful melody becomes. Although many would consider it out of character, some of these romantic outbursts occur in such early works as the ***First Piano Concerto*** and the ***First and Second Piano Sonatas***, they are more and more prevalent in his second period, as in the second movement of ***The Piano Sonata no.7*** (Minturn, 1997b, pp. 74, 80, 88). During his later years in this way his fiery youth began to mellow, but was never too distant to recall.

## General stylistic analysis

Whether in the first period or the second, certain compositional traits remained static.

Prokofiev’s style was neo-classical in theory and form, although he avoided that label (Krebs, 1970l, p. 144). In form, as mentioned earlier, he believed that Sonata Allegro, binary and ternary forms to be elastic enough to do anything one might need in music. He did not have the attitude that time had made them obsolete, as most modernists had declared. He was convinced that originality was possible within the confines of traditional musical structures (Ward, 1970, pp. 313-317). At times, he would compose one movement works employing sonata form in imitation of nineteenth century composers (Minturn, 1997c, p. 80). It is apparent that even though Prokofiev found harmony classes with Lyadov cumbersome and bland, it is clear this instruction kept his modernism in check and etched classical ideals on him for a lifetime (Prokofiev, 1979m, p. 96. 131).

Prokofiev felt that it is essential for a composition to be constructed on a tonal center, because it is like building on a rock, while having no tonal center is like building on sand. He sincerely believed that there was no future for atonal or chromatic music. In his mind, diatonic music provided greater compositional possibilities (Nest’ev, 1960l, p. 479). His harmonies were triadic, although at times polytonal or polychordal and his use of chromaticism almost verged on pitch class structure. He altered all tones of the scale, which at times lead to an expanded 12 note scale. The use of chromatic alterations led to volatile modulations.

Despite these modulations, tonic was supreme in his music and the related cadence was a must, but the resolutions were often be colored with unresolved sevenths, seconds, and tritones. He believed that dissonant combinations are independent chords which do not necessarily need to be resolved (Bakst, 1966c, p. 301). Prokofiev believed that the tonic is impulsive. He did not believe that tonal music was obsolete and felt that it could still be exciting and moving. He did not agree that evading tonic would produce melodic tension. In fact, he thought the opposite. In his view the tonic should be supreme, and treating it thus promotes natural dynamics and mobility without stagnation (Bakst, 1966d, p. 300).

He used the seven note scale most often in the context of traditional major and minor tonalities. On rare occasions he employed the use of octatonic scales, but more often used whole tone structures (Minturn, 1997b, pp. 74, 80, 88). His favorite device was a diatonic scale with a lydian fourth or a tritone and it was many times spelled enharmonically as with some of his other more common devices, which were an augmented tonic, fifth, or seventh. The intent was to use an augmentation as a new leading tone, which would propel the tonality into another key at any point. This led to an expanded scale of up to twelve notes. With this type of scale, there is no such thing as a distantly related key. The use of the augmented tonic and fifth was reminiscent of Richard Wagner. He was a favorite of Prokofiev, but it was Schubert that he came to appreciate thanks to the persistent demands of Esipova. As a result, Schubert became the greatest inspiration to Prokofiev and he often emulated Schubert’s capricious modulations (Bakst, 1966d, p. 300).

At times, Prokofiev used unrelated tones merely to add color and not for the purpose of modulation. He also used these altered tones to strengthen otherwise bland melodies. In melodies, this type of usage enhances the restatement of a theme. For instance, the first statement may be straightforward, then the second statement is given greater emphasis or excitement by adding accidentals. In doing this, every melodic note is a tonal center and tones grouped nearby are related (Nest’ev, 1960l, p. 479). This usage leads us away from harmonic devices into the realm of melody.

In melody, as Lyadov had taught him, he strove for the classical ideal of more melody with greater clarity and impact while employing thinner less important harmonies. Many times his harmonies were more polyphonic than chordal, since he used internal harmonies that overlapped as accompaniment. Other times, in keeping with a Russian folk style, he employed quartal, quintal or octave voice doublings (Bakst, 1966d, p. 300). Instead of using genuine Russian folk melodies, which he had heard as a child, he copied style instead of content. He recollected in his diary, “I was annoyed by the ‘squawking’ of the village singers -- I never listened closely to those songs and didn’t remember a single one. It is possible, of course, that I absorbed those songs despite all that. At any rate, some twenty-four years later when I first tried to make my music Russian, the material – my own, but in the Russian mood – came easily and naturally (Prokofiev, 1979n, pp. 31-32).”

His melodies were in the classical structure and were usually in four or eight bar phrases. His tendency in this direction was developed during his childhood lessons with Glier, another classical proponent like Lyadov. These early childhood experiences must have implanted an innate musical vocabulary, as much as a child’s speech patterns are embedded in the mind at an early age. His first exercises were to write small songs in 4 bar sections. In his diary, he identified this as a harmful influence which he didn’t overcome until he was older. As he recounted: “The four-bar structure makes for order; but if an entire long piece is built on 4 + 4 + 4 + 4, that order becomes intolerable and 4 + 5 is like a breath of fresh air (Prokofiev, 1979o, p. 37).” He also fell victim to the overuse of sequences, which he later came to despise. His sequences, when used, are always altered in some way to make them unique, and are never repeated more than once.

The final element in his style of melodic development, which was a favorite of Prokofiev’s in piano and orchestral settings, involved jumping around between all registers. It is very evident in the ***Children’s Pieces***, Opus 65. In all his works, he used tremendous economy of means and passed the melody from one tessitura to another. First it would appear in the bass, then a middle register and then in the higher tessitura (Bakst, 1966e, p. 302). This added color, but it was never allowed to become boring. As we evaluate these ‘stock’ compositional devices, it becomes quite obvious that a variation or surprise will appear which inevitably keeps the listener off balance (Nest’ev, 1960m, p.475). As was mentioned, unrelated tones are added for effect, along with leaps, distortions, and exaggerations, which he used to emote a particular feeling or paint a picture. Much of Prokofiev’s music, especially the works for children, is tone painting, as will become evident as we inspect the piece which is now our current focus (Nest’ev, 1960n, p. 476).

## Examining OPUS 65

The centerpieces of the second period, or stylistic shift, were: opus 64, ***Romeo and Juliet***, his pieces labeled ***Children’s Pieces***, opus 65, ***Three Children’s Songs****,* opus 68, and opus 67, ***Peter and the Wolf***. They marked a great maturation of style. He did love children and had two of his own. He spoiled them much like he had been. It was for them and his ideology of light serious music for the masses that he composed these pieces and not for punishment for ideological infractions as suggested by Krebs, at least not at this stage. In later years, however, he would use this same avenue, working with children, for enjoyment, but also to debut works that would never be performed otherwise, using what was to have been punishment to promote what would have otherwise been silenced (Krebs, 1970e, p. 163). It was also a retreat from what were tumultuous times for modern composers in Soviet Russia and a respite from completing two major works in a short period.

Robinson Harlow states that above all else, “According to Soviet cultural ideology, children were almost the most important audience for the arts. They were the hope of the communist future. As a result writers, filmmakers, directors and composers were strongly urged to create works that addressed them, for art could be used to educate children in the ideals necessary for the creation of a strong Soviet state (Robinson, 2002b, p. 306).” Prokofiev was the perfect artist to successfully approach this task. Despite his sometimes caustic, tactless and downright rude demeanor, he also had a charming childlike innocence and thirst for life (Robinson, 2002c, p. 308).

According to Nest’ev, he had a “…guileless spontaneity and purity of feeling… [and] … a vivid, uncanny remembrance of childhood (Nest’ev, 1960o, p. 457).” He loved fairy tales, Russian folklore and epic adventure. He had a unique understanding of what appealed to children, which became apparent in other pieces such as: ***Cinderella***, ***The Ugly Duckling***, ***On Guard for Peace***, ***Fairy Tale*** and ***The Stone Flower*** (Nest’ev, 1960o, p. 457). And, he always had a knack for appealing to his “market (Robinson, 2002d, p. 307).” His ***Music for Children*** was such a great success that he later transcribed it into a children’s suite for small orchestra entitled, ***A Summer Day***. In Robinson’s biography, he compares the ***Music for Children*** to ***Suggestion Diabolique*** or ***4 Pieces for Piano,*** opus 3. He admits they are less dissonant, and not as difficult in harmony and text, but asserts that some of them are just as ‘accomplished’ as the aforementioned pieces. He particularly likes ***Tarantella*** and the impressionistic quality of ***The Rain and the Rainbow*** “…in which dissonant clusters of major seconds alternate with scales and chords in “bright” C major (Robinson, 2002d, p. 307).” It is a tone painting. I believe that the tapping eighth note rhythms and chord clusters represent raindrops. The tonal movement of the piece is akin to an arch and may be a representation of a rainbow.

So, though it is not first in the collection, now that we have begun dissecting music, it is only fitting to continue with ***The Rain and the Rainbow***. Due to copyright restrictions I have had to omit musical examples. Please follow along with score in hand. Although the tonalities are complex for children’s music, each piece is extremely short and my analysis is sequential and broken into sections, so it should be easy to compare my comments to the score.

As stated, Prokofiev was masterful in his design of these small works. He knew how to make them simple yet interesting. They are each pictorially, melodically, and harmonically rich, a difficult task to achieve in such tiny works of art. The Rain and the Rainbow is one page in length, but it is rich in features.

At first glance, it is apparent that this selection is in rounded binary form (Prokofiev, 2000)^b^. It begins with section A, comprised of a 4 + 4 phrase, with contrasting periods. It is followed by a B section employing a 4 + 4 phrase with parallel periods. To round out the form, Prokofiev used a partial representation of the first theme in variation, which he ends with a six measure phrase for added flair.

In section A, measures 1–8, the key is C major, his favorite. However, his first order of business is to alter tonic up a half step to throw the listener off balance, and he uses a cadential succession in distant harmonic relationships. The harmony is chromatic and not always functional. There are non-diatonic tones used for color. The key relationships are not always certain, although the chords appear to flirt with D major and G flat minor. This is a tritone relationship, functioning like an unusually constructed six chord resolving to the dominant with the dominant returning to tonic at phrase end. This is another favorite in Prokofiev’s bag of tricks, which serves to raise the tonic, which acts as a leading tone pointing toward an implied D major, which in turn acts as the subdominant of the key of G major, resolving there, and then resolving further to C major.

This is followed by a chain of cadences, which drives the key from G flat minor, a tritone relationship to tonic, with E flat acting as a flat six, an enharmonically augmented fifth again acting as a six chord, which then resolves to G major and returns to tonic. There are four measures, which are driven by plagal cadences, which end with authentic cadences. All sequential material is varied to avoid repetition. These serve a dual purpose, they appear to be chord clusters and exude all the color of them, yet we feel the underlying driving force of cadential resolution.

Section B, measures 9–16, commences with traditional progressions and meandering scalar motion. The melody is comprised of chord tones and passing tones. The harmonies are in C major moving from tonic to subdominant, then six minor to D major which serves as dominant. Then, Prokofiev throws in a little tritone, which moves the piece into G major. This works to substitute dominant for tonic, which finally elides into the second period using an authentic cadence, which moves back into C major. The first four of the final six bars are a sequence of the first two bars of the A section and follow the same key relationships. The last two bars are like a small coda with a spicy little cadence. This piece, as with all those in this collection, moves through every tessitura so the whole keyboard is explored.

Let’s move backward toward the front of the collection to evaluate ***Regrets***. It is selection number five in the collection. It is a hauntingly melancholy composition with a depth of emotion almost too mature and complex for a child to identify with and perform effectively. It seems a pity to analyze that kind of emotional expression. Nevertheless, it is in ternary form with a coda. The whole of section A, measures 1–16, uses two eight bar phrases in four bar contrasting periods. The melody is varied from high register to low register with quickly changing dynamic ranges. The first phrase begins in the key of D minor and moves into the next phrase using the C sharp of a cadencing A major chord as a leading tone into resolution. Suspensions are used to prolong the cadence and the resolution elides into the beginning of the second phrase. The piece effectively uses the raised seventh of the harmonic minor.

In the second eight bar phrase, beginning with measure 9, there is melodic doubling in a two octave range like the kind reminiscent of Russian folk music. The melancholy nature of the melody is similar as well, but in my opinion more profoundly so. The beginning four bars of the second phrase are a variation of the melody and harmony of the beginning of the first phrase with a doubled countermelody. The second half of the phrase is almost identical to the corresponding portion of the first, except the cadence is extended and the suspensions more accentuated. To round out this section the resolution is delayed by a dramatic pause, and, as a surprise, the resolution pivots from the A in the bass in measure 16 away from tonic to the subdominant of the relative major tonality, which serves to begin the B section.

Section B, measures 17–24, has a deep chromatically ascending bass line with a high pitched descending arpeggiated right hand accompaniment. This center section is only one 8 bar phrase. It moves from the subdominant of the relative major to tonic in first inversion. The bass line, carrying the fifth, is augmented according to true Prokofiev style. It moves the tonality to G minor momentarily. Then from a raised C sharp and a passing tone, in measure 24, the second half of the center section moves to E flat major, the subdominant of B flat major. Prokofiev’s next move takes the piece into F minor (parallel minor of the relative major). Then after passing through neighbor tones, anticipations and escape tones, we reach the outline of an F minor tonic triad in which the fifth is augmented, acting as a leading tone to the dominant of D minor, the cadence of which elides with the second A section and return to home key.

Here, in the final section, measure 25, the melody is the same as in the beginning, but this time with embellishing tones, arpeggiated triads, and melodic doublings of an octave and a third. The harmonic progressions are identical. The second phrase, measure 33, in this last section has a very similar initial four bar phrase, which reaches its climax at forte and suddenly breaks off into a modulating coda with a dynamic marking of piano in measure 38. This time the central modulating tone of D flat takes us to a D, anticipating an arpeggiated inversion of B minor. The ending is approached through D minor, G minor, and back to D minor. This work touches my sensibilities. It is emotionally stirring music and a technical challenge for a young student as well.

Once again, let us move forward to the first piece in the collection, and the final piece I have chosen for analysis, which is ***Morning***. Again, as with all the compositions in this collection, it is a tone painting which evokes a picture. ***Morning*** is bright, but the tempo marking indicates tranquil. The dynamic markings range from mostly piano and pianissimo to a single mezzo forte around the climax of the piece, as the bright, tranquil sun rises and transitions into day. As the pianist journeys through the expanding dawn, Prokofiev encourages him or her to explore every tessitura of the keyboard again. Crossing hands is required several times as it is in most of the pieces.

The structure is rounded binary with an expansive and lyrical cantabile B section. The A section, measures 1–8, is an eight bar phrase with two four bar parallel periods. The incidence of multiple sequences is something highly unusual for Prokofiev since, as he mentioned, he thought they were too predictable. Instead of a sequence, it may be viewed as a motive with slight variations. Perhaps it is meant to sound like a rooster crowing at sunrise. Most everything in this section is diatonic to the key of C major. There are only a few accidentals used as neighbor or passing tones to add variety and color.

Section B, measures 9–23, is very interesting and we see Prokofiev passing the melody from bottom to top as the sun rises into the sky. There is a sparkling, arpeggiated accompaniment in the right hand and an ascending bass line moving by step. Still most everything is diatonic except the tritone in the bass in measure 11 and the raised tonic in measure 12, once again two of his favorite intervals. The raised tonic propels the piece into modulation in measure 13 and the first key felt is D major and a cadence in that key, but the return to C major is quick on a shared relationship. As we cadence on the dominant to move into the cantabile portion of the center section at measure 18, we experience the chromaticism of the tritone in measure 16.

The cantabile section is full of momentary tonal shifts that shimmer like the burning sun. Each one moves on a raised tonic, subdominant, or dominant. The richness of this type of harmony is so unique that it is rarely found in children’s collections. After the beautiful modulations, a suspension and cadence on dominant lead back to a partial repetition of the A section in measure 24. To leave the sun hanging and glittering in the morning sky, the music ends with a MM7, a double suspension that leaves an unresolved seventh.

These charming compositions deserve a more prominent place in the world of piano pedagogy. Not many piano teachers are aware of them, but that is a shame, because music so captivating and colorful is unusual in early piano literature. While beautiful, undertaking these is not without challenge to the student or teacher. There are numerous technical and aesthetic hurdles to jump. But the benefits of facing these challenges pay off in technique, evenness of tone and touch, improved dynamics, phrasing, and articulation.

The whole keyboard is explored from bottom to top, hand over hand, and it gives the student comfort in traversing the entirety of the instrument and all the nuances of each register. It challenges the young musician to learn to subordinate harmony and elevate melody and to teach him or her that it takes special effort to do it properly in those different tessituras while maintaining clarity and continuity. Alternating from clef to clef so often teaches watchfulness and quick reaction time.

Aside from the technical difficulties, teaching a child the life story and struggles of the composer and his motivating forces along with his intentions are essential to a proper performance of any music and can encourage the student as well. He or she learns to realize not only the composer’s intent, but that even the great musicians had difficulties to overcome. The student learns that genius can take shape in spite of shortcomings and that hard work and dedication pay dividends in success, even if it is slow in arriving.

In addition, the theoretical and formal analysis of a work are essential to knowing key points of transition and breathing within the structure, in fact, making the structure apparent to the performer and as a result enjoyable and identifiable to the listener. Knowledge of these things allows him or her to know when to push or pull, shape, and bring cohesiveness to their music making.

When assigning any of these pieces to a child, or to a beginning adult, it would be helpful to encourage the student to keep the arms light and as free of tension as possible. Depending on the size of the individual, the range may require altering the position at the piano to accommodate enough stretch to reach the ends of the keyboard, if it is a smaller person. If it is a larger person, it would be advisable to sit back to keep the elbows from inhibiting motion and crowding against the body. The waist should be loose and act as a pivot, so there will be no difficulty reaching the extreme ends of the piano. The feet placed properly as stays will keep the leaning pianist on the bench and off the floor. All of this will keep movements quick from one section of the keyboard to another, whether it is crossing over or jumping up or down two octaves. The gentleness of this light and free movement is required so the impact on the keys will be light and the sounds singing and not hammered, although hammered is sometimes Prokofiev’s intention. The arm motion will assist in making a smooth longer line and in aiding the shape of the phrase. This type of free arm movement will aid the hand in small rolling motions on a pivot finger to make a better legato.

Speaking of the legato touch, in order to promote clarity and deter muddy performance, the pedal should be avoided at first. It can be added once the greatest level of legato can be achieved with the arms and hands and fingers. At times, it is helpful to practice a legato section with a light, lifting staccato to maintain the lightness. Sometimes practicing legato can lead to pushing too deeply into the key bed and the sounds become too heavy for these small compositions. So, possibly alternating to a light staccato every few runs through a phrase, might keep the legato touch soft.

Legato is an important feature in this collection, since the gorgeous singing lyricism of many of the selections require a long and beautifully shaped phrase. Legato cannot be realized without smooth fingerings and resultantly the phrase cannot be properly shaped without proper legato. Prokofiev has been sparse with his fingerings in the original manuscript. The student and teacher should determine the ideal fingerings for the individual and not stray from them. More importantly in these works, such a great amount of keyboard movement requires development of physical memory in order to get exact location. Training what finger jumps where will quickly aid this.

Tempo, and a steady one, is important in Prokofiev’s music. He was known for his strict driving tempos and rhythms with sparse use of the pedal. So, in order to be true to the composer this means not over-exaggerating the lyricisms or ritenutos and the like. Maybe creating a mental picture of Prokofiev’s brusque persona might help in this. A steady metronome setting while practicing, beginning slowly and gradually increasing to the desired tempo, would be advantageous and then practicing at the chosen tempo for a while without variation would be a good idea. Once the student’s internal rhythm has developed, then begin to add breaths and hesitations and the marked effects. Push and pull only slightly. As I recall, according to some recordings of Prokofiev playing his own music, by our standards, he isn’t as strict as we would expect, but by 19th and early 20th century standards he was very strict (Prokofiev, Prokofiev Plays Prokofiev: piano concerto no. 3/Visions Fugitives, 2001). So, we should keep that same spirit of proportion in relation to our modern day practice as long as the feeling or heart is not lost in the process.

Dynamics and articulations should be adhered to strictly. Most of the effect of these works would be lost without proper attention and execution of this facet. But always determine what the melody is and raise it one dynamic notch above everything else. As we have learned, Prokofiev is a neo-classicist. In the classical style of music, the melody is supreme and should stand out while the harmony seasons it lightly. Matching this style, the same should be done with pedal. The only exception to this neo-classical rule might be harsh articulations and larger crescendos and diminuendos. While those were not the rule in the classical era, they are a development of modern music and are retained, and at times when called for, are exaggerated in the world of Prokofiev.

The elements of this paper show an all-encompassing pedagogical approach that motivate the teacher and the student to work together in researching their performances and gaining maximum benefit from not only this volume of compositions, but each subsequent effort as well. Always study the biography, the style, the cultural milieu at the time during the composer’s life, the overarching stylistic tools of the artist spanning his lifetime, and then dissect the music for form and harmony. Then finally, approach the execution at the keyboard logically, thoughtfully, and artistically. The performance will blossom as a result and the reception of the listener will improve. Everyone benefits!

## Endnotes

^a^Izrail’ Vladimirovich Nest’ev. This quotation is from a secondary source. I had difficulty finding this issue of *The Musical Observer* published in November 1918. I understand that quoting secondary sources is frowned upon, although not completely forbidden when the item is out of print. I found this quote significant. It shows Prokofi ev’s honesty. It might be encouraging to talented students struggling with technique to read this. Paraphrasing would ruin its impact. It is also significant in that Prokofiev indirectly admitted that his technique was not sufficient to have won the Rubenstein piano competition on his pianistic merits alone as we discussed in the preceding section. I hope the reader will indulge my decision.

^b^Prokofiev’s *Music for Children*, Opus 65, published by Kalmus was the score used for the musical analysis portion of this paper and is listed in the reference section. All further analysis beyond this point is the author’s.

## Consulted but not cited

Prokofiev, S. (1990). Horowitz plays: Prokofiev, Barber, Kabalevsky sonatas [Recorded by V. Horowitz]. [CD]. RCA Victor.

Prokofiev, S. (1993). Complete music for solo piano [Recorded by G. Sandor]. [LP]. Vox.

Prokofiev, S. (1994). Piano sonatas nos. 2, 7, and 8 [Recorded by B. Glemser]. [CD]. Naxos.

Prokofiev, S. (1996). Romeo and Juliet [Recorded by M. T. Thomas]. [CD]. RCA Red Seal.

Prokofiev, S. (1999). Piano sonatas nos. 1, 3, and 4 [Recorded by B. Glemser]. [CD]. Naxos.

Slonimsky, N. (2000). *The Great Composers and their Works.* New York: Schirmer Books.

Thompson, K. (1973). *A Dictionary of Twentieth-Century Composers (1911–1971).* New York: St. Martin's Press.

## Bibliography

Bakst, J. (1966). *A History of Russian-Soviet Music.* Westport: Greenwood Press.

Krebs, S. D. (1970). *Soviet Composers and the Development of Soviet Music.* New York: W.W. Norton.

Minturn, N. (1997). *The Music of Sergei Prokofiev.* New Haven: Yale University Press.

Nest’ev, I. V. (1960). *Prokofiev.* Standford: Standford University Press.

Prokofiev, S. (1979). *Prokofiev by Prokofiev: A Composer's Memoir.* Garden City: Doubleday.

Prokofiev, S. (2000). *Children's Pieces, Opus 65.* Boca Raton: Kalmus.

Prokofiev, S. (2001). Prokofiev plays Prokofiev: Piano concerto no. 3/Visions Fugitives [Recorded by S. Prokofiev]. [CD]. Naxos.

Robinson, H. (2002). *Sergei Prokofiev: A Biography.* Boston: Northeastern University Press.

*The New Grove Dictionary of Music and Musicians* (Vol. 15). (2001). New York: Grove's Dictionaries.

Ward, W. R. (1970). *Examples for the Study of Musical Style.* Dubuque: W.C. Brown Co.
